# Advance care planning in people with intellectual disabilities, more than ‘to resuscitate or not to resuscitate’: A qualitative interview study with general practitioners and family caregivers

**DOI:** 10.1080/13814788.2026.2745929

**Published:** 2026-10-19

**Authors:** Marnix De Grim, Jakke Hauman, Oliwia Zmysłowska, Sophie Opsomer, Johan Wens, Anke Van Dijck, Annelies Colliers

**Affiliations:** a Primary and Interdisciplinary Care Antwerp Research group (ELIZA), Faculty of Medicine and Health science, University of Antwerp, Antwerp, Belgium; b Department of Rehabilitation Medicine, AZ Klina hospital, Brasschaat, Belgium

**Keywords:** Advance care planning (ACP), intellectual disability, family caregivers, general practitioners, qualitative research

## Abstract

**Background:**

Advance care planning for people (ACP) with intellectual disabilities requires timely and carefully communication between family caregivers and general practitioners.

**Objectives:**

Despite its recognised importance, little is known about how these stakeholders experience advance care planning and decision-making processes, particularly regarding goals of care and do not resuscitate (DNR) orders, highlighting a critical gap in ensuring person-centred palliative care for this population.

**Methods:**

A qualitative design was used to investigate how Belgian general practitioners and family caregivers of adults (≥18 years) with moderate to profound intellectual disabilities (IQ ≤ 50) approach and experience decision-making about treatment goals and DNR directives. The data were analysed thematically.

**Findings:**

Interviews with six family caregivers and five general practitioners identified six themes: limited preparedness for ACP, the need for open discussion, communication challenges, involvement of key stakeholders, timing, and positive outcomes. Participants reported limited ACP knowledge and a need for earlier, broader conversations that extend beyond DNR decisions. Emotional barriers, uncertainty, and concerns about discussing end-of-life care too early often delayed ACP. Family caregivers generally expected GPs to initiate discussions, while both groups emphasised multidisciplinary involvement and timely, proactive conversations. Despite challenges, ACP discussions were often experienced as reassuring and helpful.

**Conclusions:**

ACP for adults with moderate to profound intellectual disabilities is shaped by knowledge gaps, communication barriers, and uncertainty about timing. Early, proactive, and multidisciplinary approaches that address personal values alongside treatment preferences may support person-centred decision-making and improve experiences of end-of-life planning.

## KEY MESSAGES

- Advance care planning for adults with intellectual disabilities is challenged by knowledge, communication, stakeholder involvement, and timing.
- Family caregivers and general practitioners valued early, open discussions beyond do not resuscitate decisions, addressing care goals and personal values.
- Future research should optimise involvement of individuals with intellectual disabilities and their caregivers.

## Introduction

Intellectual disability affects approximately 2–3% of the general population and is characterised by limitations in intellectual functioning (e.g. learning, reasoning) and adaptive behaviour (e.g. communication, independent living) [1]. Severity varies widely, and intellectual disability may occur as part of a syndrome or as an isolated condition [2,3]. Advances in healthcare, and living conditions have improved life expectancy and quality of life [4]. Nonetheless, palliative care and end-of-life decision-making remain critical considerations for this population.

Advance care planning (ACP) enables patients to discuss their preferences, goals, and wishes regarding end-of-life care anticipating a time when independent decision-making may no longer be possible [5]. ACP includes discussing personal values and goals of care, preferences regarding life-sustaining treatments, preferred place of care, and the involvement of family members or proxy decision-makers in future medical decisions [6]. Decisions regarding cardiopulmonary resuscitation, such as do-not-resuscitate (DNR) orders, represent one specific component. Effective communication regarding these issues is essential to ensure that patient preferences and those of their proxies are respected.

While ACP is well studied in the general population, much of this knowledge cannot be directly applied to individuals with intellectual disabilities. Literature highlights the importance of initiating ACP discussions early and in a way that accommodates the unique needs of individuals with intellectual disabilities and their family [7,8]. People with intellectual disabilities often experience multiple comorbidities and face communication challenges, complicating the assessment of needs and preferences, and the evaluation of treatment impact, particularly in individuals with moderate to profound intellectual disabilities [9,10]. However, evidence indicates that individuals with intellectual disabilities are less likely to have DNR orders in place, increasing the likelihood of resuscitation compared to those without intellectual disabilities [11]. Moreover, for half of the deceased individuals with intellectual disabilities in one study, the first documentation of palliative care occurred less than one month before death suggesting that end-of-life discussions often occur too late to inform care adequately [12].

Individuals with intellectual disabilities should be involved in ACP discussions. Some people with mild to moderate intellectual disabilities can participate meaningfully in conversations about their future care when appropriate support and communication strategies are used [8]. However, they are still insufficiently involved in such discussions in practice [7,8].

Due to cognitive and communication limitations, individuals with more severe intellectual disabilities may have limited opportunities to participate directly in ACP discussions, which places greater responsibility on family caregivers and healthcare or social professionals [8,13]. The care of people with intellectual disabilities often involves a multidisciplinary network of professionals, including social care staff who may have long-standing relationships with individuals and detailed knowledge of their daily lives, and therefore can be involved in such conversations [14–16]. For children and adolescents, paediatricians, hospital specialists, and parents or legal guardians are primarily involved in these decisions [17].

In the Belgian context, as in many other Western primary care systems, patients often rely on their GP as the first point of contact for medical decision‑making, including discussions about future care [18,19]. However, the organisation of care and the central role of GPs in ACP can vary by country and healthcare system [20–22]. Moreover, there is an international shift towards interprofessional collaboration in ACP [23]. Furthermore, in some countries, specialised physicians in intellectual disabilities’ medicine are involved in the care of people with intellectual disabilities [24,25].

The present study focuses on adults with moderate to profound intellectual disabilities, as decision-making about future care in this population often involves complex communication processes and surrogate decision-making. We focused specifically on general practitioners and family caregivers, as they are typically closely involved in medical decision-making related to ACP within the primary care context.

This study explores how ACP and DNR decision-making in individuals with moderate to profound intellectual disabilities are understood, approached, and experienced from the perspectives of GPs and family caregivers, aiming to inform strategies for timely, patient-centred end-of-life care.

## Methods

### Study design

This qualitative study used individual in-depth interviews and inductive reflective thematic analysis. Reporting followed the COREQ criteria and is provided in Supplemental Material 1.

### Participants

Two groups of participants were included: GPs and family caregivers of individuals (≥18 years) with moderate to profound intellectual disabilities, defined as an IQ ≤ 50. These groups were selected because both play key roles in communication and decision-making regarding ACP and DNR decisions. In Belgium, when individuals lack decision-making capacity, healthcare decisions are made by a court-appointed representative.

Although their roles and perspectives differ, including both groups allowed us to explore the ACP process from complementary viewpoints and to better understand the barriers and facilitators influencing these discussions. Separate semi-structured topic guides were developed for GPs and family caregivers to reflect their specific experiences while maintaining consistency across interviews. During analysis, attention was paid to both shared themes and potential differences between the two participant groups.

### Recruitment and sampling

Participants were primarily recruited through the researchers’ professional networks and relevant patient organisations. To capture diverse perspectives, purposive sampling was used to enhance heterogeneity within the study population with respect to participant age, gender, professional experience (for the GPs), and type of caregiving relationship. Participants were selected based on their direct experience with ACP and DNR decision-making for adults with moderate to profound intellectual disabilities. Given the specificity of these experiences and the in-depth interviews, the aim was to obtain rich data rather than a large sample size. Thirty-four candidates received an invitation letter outlining the study and voluntary participation. The interviewers did not have prior relationships with participants. Participants were assured of confidentiality and pseudonymisation. Data sufficiency was reached after eight interviews, when no substantially new codes emerged. Three additional interviews were conducted to further explore variation and refine the emerging themes, resulting in a total sample of 11 participants. Given the exploratory nature of the study, analyses were conducted across the full dataset to identify shared and contrasting perspectives.

### Data collection

Written informed consent was obtained. Demographic information (age, gender, professional experience, and type of disability) was collected at the start of each interview. Interviews were conducted individually, either in person in their own home or GP practice, or *via* Microsoft Teams, and lasted 30–60 min. Two semi-structured interview guides were developed for GPs and family caregivers, with overlapping questions. The guides were informed by the literature and refined through discussion with the team of experienced qualitative researchers. Both interview guides are provided as Supplementary Material 2. Interviews were audio-recorded and transcribed verbatim. The research team did not return transcripts to participants for correction, and no repeat interviews were conducted. Data were pseudonymised and stored securely. NVivo software was used for data management and coding.

### Research team and reflexivity

Interviews were conducted by three final-year medical students as part of their master’s thesis for the degree of medical doctor, one student conducting an interview and roles rotating across interviews. To enhance familiarity with the data and ensure analytical rigour, each interview was transcribed by one of the other two student researchers and subsequently reviewed by the remaining third student. This approach ensured that all three authors were actively engaged with each interview throughout the analysis process. Qualitative research is part of the medical curriculum of the students. Furthermore, prior to data collection, the students received a half-day training in qualitative research methods, including interviewing techniques and data analysis.

The research process was supervised by AC (GP, PhD) and AVD (MD, PhD), experienced researchers, with expertise in intellectual disability care. JW (MD, PhD), a professor of primary care and palliative care, and SO (MD, PhD), a GP and palliative care researcher. All supervisors were experienced qualitative researchers.

Reflective notes were maintained throughout the study. Regular research team meetings were held to discuss emerging codes and themes and to support the development of the final thematic structure.

### Data analysis

The first three authors repeatedly reviewed the transcripts and conducted an inductive thematic analysis following Braun and Clarke [26]. They double-coded the interviews, discussing coding to reach consensus. The first four transcripts were additionally double-coded by the supervising researcher, and all transcripts were reviewed by this researcher. Codes were iteratively reviewed and discussed in regular team meetings to identify patterns and resolve discrepancies. Similar codes were grouped into categories and overarching themes, integrating barriers and facilitators into an initial framework after 5–6 interviews. Subthemes were combined into overarching themes when they captured related concepts, and relationships between themes were examined by identifying patterns and contrasts across participants. The framework was refined through iterative analysis and discussion to ensure coherence and grounding in the data. Finally, the key themes were selected by consensus. Preliminary findings were also reviewed by two additional experts (JW and SO) in qualitative research and palliative care to ensure rigour and grounding in the data. Representative quotes were selected to illustrate the identified themes, maintaining a strong connection to participant perspectives.

## Findings

Out of 34 individuals invited to participate, 11 agreed to be interviewed, including six family caregivers and five GPs. Reasons for non-participation were systematically collected: 20 invitees did not respond, and among those who provided feedback, time constraints and limited experience with the target population were cited as reasons for declining participation. Table 1 presents the demographic characteristics of the participants. Table 2 presents the subthemes that were combined to form each overarching theme. Thematic analysis of the interview data resulted in the identification of six major themes, which are described in detail below [1]: limited preparedness and understanding of advance care planning [2], the need for open discussion [3], challenges in communication [4], the importance of involving key stakeholders [5], the role of timing, and [6] positive outcomes of these conversations.

**Table 1.** Participants’ demographics.

	GP	Family caregiver
**Sex**		
Male	1	1
Female	4	5
**Age (years)**		
Average	45.2	60.3
Min	30	46
Max	79	70
**Experience (years) with individuals with ID**		
Average	16.8	
Min	4	
Max	46	
**Place of care**		
Residential setting		5
Ambulatory setting		1

Residential setting refers to services that provide housing and daily support for people with intellectual disabilities. Ambulatory setting provides outreach or community-based support to individuals living independently or with their families. However, this distinction is not always clear-cut, as many individuals living in residential settings regularly spend weekends, holidays, or other periods with their families and maintain strong involvement in family life.

**Table 2.** Overview of categories.

Preparedness/understanding	The need for open discussion about ACP	Challenges in discussing ACP	The importance of involving key stakeholders in ACP	Timing as a critical factor in ACP conversations	Positive outcomes
GPs: experience of ACP (e.g. elderly care, less ID) Caregivers: little to none knowledge Caregivers’ experiences	GPs experience facilitates GP is initiating Broader scope of discussions Proactively informing Space for emotions Presenting ACP as dynamic and evolving not only DNR Information and interests	Overlooked when healthy Confronting topic Caregivers expecting initiative from doctors Few contacts between GP and caregiver Uncertainty Communication difficulties Administration not adapted	The caregiver should choose who to include The (non-) participation of people with intellectual disabilities Importance of professional caregivers Knowledge of the person	Negative emotions linked to ACP when person with intellectual disabilities is healthy Informing caregivers to start thinking about ACP Set moments to explain ACP to caregivers Worsening health and QoL as initiator Facing caregiver’s own end of life Caregivers rely on GPs to initiate ACP ACP timing should be personalised	Emotional relief and reassurance Thankfulness

### Limited preparedness and understanding of advance care planning

Participating GPs had experience with ACP, but reported that existing approaches and documentation, largely derived from elderly care, did not always reflect the needs of people with intellectual disabilities. Physicians reported insufficient preparation for ACP discussions during medical training, with confidence typically gained through additional training or experience.

*“Erm, theoretically in medical school that [ACP] is touched upon a bit, but I think that only after the training you really know how concrete that is (…). In the beginning, it’s always very, (…) very unnatural, and you don’t know how to bring it up. So in medical school it was addressed, but to a lesser extent,…” – GP 9*

However, such training rarely focuses on people with intellectual disabilities.

*“You did learn how to have conversations, but the target population (people with intellectual disabilities) was unknown to you. That, that I only got to know by starting to work in a facility for people with an intellectual disability” – GP 1*

Family caregivers had limited formal knowledge of ACP despite relevant experience with ACP-related decisions. This may contribute to uncertainty about when and how to initiate ACP, partly explaining why discussions are delayed or avoided and why GPs often take the lead.

### The need for open discussion on ACP

Family caregivers expressed a strong desire to gain more information about ACP. They voiced a preference for receiving instructions about ACP proactively, rather than only when immediately relevant, for example, in palliative or critical life-or-death situations. One caregiver described the distress of being confronted with ACP during an emergency admission.

*“And suddenly they ask me, ‘Does she have a DNR code?’ I say, ‘Oops, what’s a DNR code?’ and there I was” – CG 8*

*“I think that if you, (…) as a doctor, know that they [the family caregivers] will once be faced with that [end of life] decision, then it’s okay for me, even as a parent, that you’re confronted with it early on. Because that is also a process, to be able to make that decision (…).” – CG 8*

Informative sessions for family caregivers were suggested as a valuable first step to engage in conversations about the topic and to provide an address questions.

*“When I first gave an evening information session for parents, afterwards I had a lot of people who came to ask questions about their family’s ACP.”* – *GP 1*

The GPs agreed they carry out an important informant function during both informative sessions as well as one-on-one discussions. A physician’s adequate experience and their willingness to engage in these discussions proved to further ease family caregivers into participating in ACP conversations.

*“So we started making plans… and checked with the family: ‘Are you all right with this?’ Most of them were extremely grateful, as they had often been thinking about the same issues but hadn’t dared to ask.” – GP 7*

Family caregivers and GPs emphasised the importance of broadening the scope of ACP, noting that discussions should extend beyond DNR orders to include broader life values, family preferences, and religious beliefs. Furthermore, GPs and family caregivers both noted that framing ACP as a dynamic, evolving process rather than a fixed decision oftentimes reduces anxiety around this topic.

### Challenges in discussing ACP

Family caregivers reported several challenges to engaging in ACP. Firstly, starting an ACP discussion is difficult for the family caregivers due to the uncertainty about the future of the person with intellectual disabilities.

*“That is also the problem, not knowing that in advance. How someone is, what is going to happen, or what the outcome might be.”* – *CG 6*

Family caregivers wished to be better informed about the typical disease course, suggesting that doctors could prepare families for what to expect.

*“Maybe the doctors could prepare [us] for it and say: ‘Look, such children usually have a very grievous ending, in which the body usually no longer responds, or the organs shut down one by one.’ And then maybe you kind of know what you’re facing.”* – *CG 8*

ACP discussions often confronted family caregivers with the mortality of their loved one, making them emotionally difficult.

Denial was reported as a coping mechanism, with family caregivers acknowledging that they tended to avoid thoughts about future care. Feelings of guilt further complicated the willingness to engage in ACP conversations. Both GPs and family caregivers noted that initiating ACP could lead to fears of being perceived as uncaring.

*“Because very often (…) the [family] caregivers, the network is afraid to bring it [ACP]) up themselves out of fear, like: ‘if I say this (…), then I might be considered a bad, uninvolved parent’.* – *GP 1*

The relatively young age or apparent good health of some individuals made ACP seem premature, even when complex medical conditions suggested that these discussions were appropriate.

*“While the persons are still healthy, I really find that very… It’s like a very financial decision, like come on, we’re not going to put in too much effort anymore. I think so… If they are of course sick and you see that the person is really suffering, then it’s more of a humane decision.”* – *CG 5*

Although GPs felt ACP could be initiated by either party, most family caregivers expected physicians to take the lead.

Physicians and other healthcare professionals should gauge the families’ readiness to discuss end-of-life decisions, encouraging but not enforcing this topic and revisiting it later if met with hesitancy from the family caregiver’s side.

*“And that [ACP discussion] might then have to come from doctors. (…) And I think maybe yes, isn’t that partly also a task for a doctor? I don’t know. That’s not something I should express myself about.”* – *CG 2*

Caregivers noted that GP visits were often brief and did not always coincide with their presence, which could limit opportunities for direct interaction and development of a trusting relationship.

### The importance of involving key stakeholders in ACP

Participants felt that individuals with intellectual disabilities should be involved in ACP only when they could understand the topic. The family caregivers indicated that, in the case of the individuals with intellectual disabilities they cared for—many of whom had severe to profound impairments—direct involvement in ACP discussions was not always seen as appropriate.

*“In an ideal scenario, of course, it would be that they [people with intellectual disabilities] are involved [in ACP discussions]. But I think that for many, it would cause distress, if that was discussed with them, because they can’t understand why or how …”* – *GP 9*

While opinions about who should participate in decision-making varied, participants consistently emphasised the importance of including those most closely involved in the individual’s daily care. This encompassed family members as well as professional caregivers such as the GP, nurses, residential care staff, and social workers, reflecting the need for decision-making to be informed by those with intimate knowledge of the person’s preferences and routines.

*“It is often discussed that advance care planning needs to be adapted for a particular person, for example when there are concerns about underlying dementia and further assessment may be needed—these decisions are typically made within the multidisciplinary team. At the same time, during the annual meeting with the family, all involved professionals are present and able to contribute their perspectives.” – GP 9*

In the absence of family members, GPs relied on professional caregivers’ expertise, as these professionals provide essential context that GPs cannot obtain through their typically brief interactions with the individual.

### Timing as a critical factor in ACP conversations

Declining health or quality of life, for example due to age or illness, can serve as a natural prompt to initiate these conversations. Similarly, the family caregiver’s or another family member advanced age or sudden illness could trigger considerations about who would assume responsibility for care after their death.

GPs expressed a desire to adopt a more proactive and standardised approach, recognising the importance of initiating ACP discussions before critical situations arise. This reflects an increasing awareness among practitioners that ACP should be integrated into routine care rather than reserved for moments of crisis, while acknowledging that determining the most appropriate timing for ACP discussions is often difficult.

*“Recently, the situation has become more urgent for those two elderly residents. But perhaps we should look into this as well and consider a conversation for the other residents too.” – GP 4*

*‘It’s congenital, so you can’t pinpoint a specific moment and say, “This is the moment you*

*have to do it.”*’ – *GP 3*

Family caregivers emphasised that they would not want to be confronted with ACP for the first time during a critical situation, as this could be overwhelming and interfere with their ability to make considered choices.

*“I don’t want to end up in that situation again where we enter a hospital, or at a moment when things are very critical, and we must make that decision or someone then comes with the question: ‘do you have a DNR code?’”* – *CG 8*

GPs working in a residential setting introduced the topic at the beginning of the stay of the person with intellectual disabilities, or at a standard time, to encourage early conversations. This approach can reduce uncertainty about the timing of conversations and frame ACP as a normal, integrated aspect of care for family caregivers.

*“And they (the family) understand that; when we say, “We want to sort everything out for everyone”, they get it.” GP 7*

### Positive outcomes of ACP conversations

Family caregivers who had previously engaged in ACP conversations reported feeling relieved and satisfied that they were able to discuss this topic. GPs likewise observed relief and gratitude among family caregivers following ACP discussions.

*“It has such a positive effect. Not just the idea of ‘what are we no longer going to do’, but also, yes, ‘what are we still going to do’, because it gets you thinking about what we ‘do’ still consider important to do.” – GP 1*

*“And they’re very happy and also very grateful when you bring it up yourself, and it also gives them a sense of reassurance, like: okay, it’s settled. As if to say: yes, it’s been agreed.” – GP 1*

Table 3 summarises the main facilitators and barriers identified in this study.

**Table 3.** Summarising table of facilitators and barriers in ACP for persons with intellectual disabilities.

Barriers for ACP conversation	Facilitators for ACP conversation
Unclear future Young age Good current health Inconsistent ACP discussion and planning Lack of contact with physician Association with negative emotions Caregivers unawareness of ACP’s existence Reliance on physicians’ judgement in critical moments Caregivers rely on staff* to initiate ACP when appropriate Staffs’* hesitancy to initiate ACP discussions Lack of ACP experience in staff* Too direct communication Divided opinions	Presenting ACP as a dynamic, evolving plan Addressing the ACP knowledge gap Exposure to ACP *via* informative sessions Integration in medical training Provides clear and structured information Actively involve key figures Recognises emotional challenges and offer support Highlighting benefit of ACP

* Staff represents all medical staff in a healthcare/social environment.

## Discussion

This study explored how family caregivers and GPs experience ACP for adults with moderate to profound intellectual disabilities. The findings show that limited knowledge, communication barriers, and emotional complexity often hinder ACP, yet open and timely dialogue can also create opportunities for shared understanding and relief. Both family caregivers and GPs expressed uncertainty about when and how to initiate ACP conversations, highlighting the need for early and proactive discussion that goes beyond DNR decisions to include broader life values and family preferences. Similar barriers have been reported, including limited consultation time, difficulties discussing treatment limitations, documentation challenges, and reluctance to start ACP in the absence of a clinical trigger [21,27].

The lack of adequate training and resources for healthcare professionals, has been consistently identified as a challenge [28–31]. Studies in primary care and community settings highlight barriers such as limited knowledge about ACP and, professional discomfort initiating conversations. Structured approaches, including ACP training, communication tools, and practice-level resources that facilitate the initiation, documentation, and follow-up of ACP conversations, may support more consistent implementation [27,32,33]. While numerous studies have underscored the insufficient preparation of professionals, less attention has been given to the limited understanding and preparedness of family caregivers [14,34]. Better guidance, education, and accessible information for family caregivers are needed, as their understanding and involvement are essential. A broader cultural shift is also needed to normalise discussions about future care outside crisis situations. ACP is often delayed in adults with intellectual disabilities, sometimes being documented only in the final weeks of life [12]. Discussions usually take place under acute circumstances, triggered by health decline rather than proactive planning, partly due to diagnostic uncertainty and doubts about patients’ capacity to participate [8]. Such crises further complicate communication and exclude individuals with intellectual disabilities from decision-making [7]. Participants described a wish for a more proactive approach to ACP discussions. Early and transparent communication, even in stable periods, can help build trust and prepare families for future choices. Participants viewed ACP as an ongoing discussion of values and preferences rather than a single DNR-focused conversation [35]. All participating GPs believed that both parties could initiate ACP, whereas most family caregivers expected physicians to take the lead. Physicians should therefore be aware of these expectations and address ACP proactively.

Family caregivers often experience guilt and a sense of responsibility when making end-of-life decisions for individuals with intellectual disabilities [7]. This may also contribute to hesitation in initiating ACP discussions, as both caregivers and professionals may fear being perceived as uncaring.

Uncertainty about disease progression further complicates ACP in this population. Symptom recognition and pain assessment are often difficult and rely heavily on (family) caregiver interpretation, while validated ACP tools and clinical guidelines remain limited [28,31].

Family caregivers and GPs considered the involvement of family members, support staff, and, where possible, the person with intellectual disabilities, essential. Discussing future care often provided reassurance and a sense of empowerment. However, participation of people with intellectual disabilities was generally considered appropriate only when they could understand the topic, reflecting findings from previous studies that report limited involvement due to perceived cognitive and communication barriers [7,8,12,15,29,30,36,37]. However, this reflects a substitute decision-making approach that differs from the UN Convention on the Rights of Persons with Disabilities, which emphasises supported decision-making and the right of people with disabilities to have their wishes, preferences and values represented, even when they cannot communicate these independently. Accessible information can enable meaningful ACP participation, although this has been demonstrated mainly among those with mild to moderate impairments [28,38]. Assessing comprehension of concepts such as death could therefore help identify those capable of participating [39]. However, ACP should not focus solely on cognitive capacity but would benefit from approaches that seek to identify and represent the person’s preferences through supported decision-making, involving family caregivers and others who know the individual well. Previous research emphasises that a strong, collaborative relationship between healthcare or social professionals, family caregivers, and individuals with intellectual disabilities is essential for fostering open communication about ACP [28,40].

### Strengths and limitations

Including both GPs and family caregivers provided complementary perspectives on ACP, enhancing strengthening the relevance of the findings.

Although data sufficiency was achieved, the sample was small and all participating GPs had prior ACP experience, while family caregivers varied in their familiarity with ACP. The use of three interviewers may have introduced variation in interviewing style and interpretation. Finally, social desirability bias cannot be excluded, as some participants may have withheld or softened certain experiences due to feelings of guilt or shame. Finally, although interviews were conducted both in person and online, the latter may have limited observation of non-verbal cues.

## Conclusion

This study highlights the challenges and opportunities of ACP for individuals with intellectual disabilities. Limited preparedness and understanding among GPs and caregivers indicate a need for targeted training, and a broader shift towards routine discussions of future care. ACP should be initiated proactively as an ongoing dialogue about individual values rather than a discussion limited to DNR decisions. Early conversations during stable periods may reduce crisis-driven decision-making, while person-centred approaches can support communication and shared understanding. Participants’ feelings of relief and empowerment underscore the value of timely ACP in promoting more compassionate and equitable end-of-life care.

## Supplementary Material

Supplemental Material

Supplemental Material

## Data Availability

The datasets used and/or analysed during the current study are available from the corresponding author on reasonable request.
