# Supplementary material for: Advance care planning in people with intellectual disabilities, more than ‘to resuscitate or not to resuscitate’: A qualitative interview study with general practitioners and family caregivers
Source: Eur J Gen Pract. 2026 Oct 19;32(1):2745929. doi: 10.1080/13814788.2026.2745929 (PMC13686196; doi:10.1080/13814788.2026.2745929)
Supplement: Supplementary file 1 [file IGEN_A_2745929_SM2435.docx]

# Supplementary material: topic guide

## Part 1: family caregivers

Knowledge and understanding of advance care planning / DNR orders

- What does advance care planning mean to you?
- Can you tell us what you already know about advance care planning / DNR orders for people with intellectual disabilities?
- Where and when did you first hear about this topic?
- Are there aspects of this topic that you feel less familiar or confident about? To what extent do you think there are gaps in your knowledge about this?
- What kind of information or support would help you learn more about this?

Experiences with advance care planning / DNR discussions, communication with physician/ Experiences and emotional aspects of conversations

- Have you already talked about advance care planning for people with intellectual disabilities with the physician?
- Why or why not?
- What prompted this conversation?
- At what stage in the care process did this conversation take place?
- Who initiated the conversation?
- To what extent do you feel comfortable or able to talk with the physician about this topic?
- How did you experience this conversation?
- What were positive aspects of this conversation?
- What could the physician do differently to make such a conversation better in the future? If another professional were to have this conversation, what advice would you give them?
- What specific topics were discussed in this conversation?
- Looking back, do you think the timing of the discussion was appropriate? At what stage in care do you think it is important to have such a conversation?

Decision-making and documentation

- Have you already documented a DNR/ABC order?
  - Why or why not?
  - How did (or would) you start this process?
- How were you supported? How did you experience it? (sufficient in your opinion?)
- What feelings come up when thinking about making or recording these decisions? Any concerns?
- With whom did you discuss this decision? Who was involved?
- How was/will be the person with an intellectual disability involved in this discussion?
  - Do you think it is important that the person with an intellectual disability is involved in such discussions?
  - How were the wishes or values of the person with an intellectual disability taken into account? How could the person with an intellectual disability be more involved?
- How did you experience the role of the physician in this discussion?
- Who else was involved? Who else should be involved?

## Part 2: General practitioners

Knowledge and understanding of advance care planning / DNR orders

- When you hear the term ‘advance care planning,’ what comes to mind for you in the context of intellectual disability? What do you know about advance care planning / DNR / ABC orders for people with intellectual disabilities?
- Where and when did you first hear about this topic? (during your medical training or later in practice)
- Are there areas where you would like to feel more confident or informed? To what extent do you feel there are still aspects missing in your knowledge about this topic?
- What opportunities do you see for further training on this subject?
- What kinds of education or support would help you feel more prepared for these discussions? Are there opportunities for specific training related to this topic for patients with intellectual disabilities?
  - Do you feel sufficiently trained to discuss these issues with patients and their family caregivers?

Experiences, contact and communication with family caregivers and patients

- How would you describe your contact with family caregivers?
- Can you describe a recent situation where this topic came up?
- To what extent do you discuss advance care planning / DNR with family caregivers?
- What differences do you experience in such conversations with family caregivers of people with severe versus moderate intellectual disabilities?
- To what extent do you discuss these topics directly with the person with an intellectual disability?
- Who usually initiates these conversations? — you or the family caregivers or other?
- What is typically discussed in these conversations?
- How do you approach this topic with people with a moderate intellectual disability?
- How do you approach this topic with people with a severe intellectual disability?
- What do you see as reasons why this topic is sometimes not discussed? Are there organizational, emotional, or relational factors that make it difficult to bring this up?
- At what stage in care do you think it is important to have these conversations in the context of intellectual disability? At what point in the care trajectory do you usually start such a discussion?

Experiences and emotional aspects of conversations

- How do you usually experience these conversations?
- What positive experiences have you had in such discussions?
- In what areas do you feel you could improve to handle these conversations better?
- How do you experience differences when it comes to patients with moderate versus severe intellectual disabilities?

Decision-making and documentation

- How does the documentation take place between you and the family caregivers?
- How do you usually start these conversations?
- How do you feel during and after these discussions?
- In what ways do you support family caregivers in making these decisions? Do you feel this support is sufficient?
- With whom do you discuss these decisions?
  - Who is involved?
  - Who do you think should also be involved?
- How is the person with an intellectual disability involved in these discussions?
  - Do you think it is important that the patient is involved?
  - How could the person with an intellectual disability be more involved?
  - How does your opinion differ when it comes to people with moderate versus severe intellectual disabilities?
  - How did you experience your role as a physician in these discussions?
