# Supplementary material for: Advance care planning in people with intellectual disabilities, more than ‘to resuscitate or not to resuscitate’: A qualitative interview study with general practitioners and family caregivers
Source: Eur J Gen Pract. 2026 Oct 19;32(1):2745929. doi: 10.1080/13814788.2026.2745929 (PMC13686196; doi:10.1080/13814788.2026.2745929)
Supplement: Supplementary file 2 [file IGEN_A_2745929_SM2436.doc]

**Suppl. 1: Consolidated criteria for reporting qualitative studies (COREQ): 32-item checklist**

Developed from:

Tong A, Sainsbury P, Craig J. Consolidated criteria for reporting qualitative research (COREQ): a 32-item checklist for interviews and focus groups. *International Journal for Quality in Health Care*. 2007. Volume 19, Number 6: pp. 349 – 357

| **No. Item** | **Guide questions/description** | **Reported on Page #** |
| --- | --- | --- |
| **Domain 1: Research team and reﬂexivity** |  |  |
| *Personal Characteristics* |  |  |
| 1. Interviewer/facilitator | Which author/s conducted the interview or focus group? | ***P.6 research team and reflexivity:*** Interviews were conducted by DM, HJ, and ZO, final year medical students at the time of the study |
| 2. Credentials | What were the researcher’s credentials? E.g. PhD, MD | ***P.6 research team and reflexivity:*** Interviews were conducted by three final-year medical students as part of their master’s thesis for the degree of medical doctor.  ***P.7*** The research process was supervised by AC (MD, PhD) and AVD (MD, PhD), experienced researchers, with expertise in intellectual disability; JW. (MD, PhD), a professor of primary care and palliative care; and SO (MD, PhD), a GP and palliative care researcher. All supervisors have extensive experience with qualitative research. |
| 3. Occupation | What was their occupation at the time of the study? | ***P.7 research team and reflexivity:*** The research process was supervised by AC (MD, PhD) and AVD (MD, PhD), experienced researchers, with expertise in intellectual disability; JW. (MD, PhD), a professor of primary care and palliative care; and SO (MD, PhD), a GP and palliative care researcher |
| 4. Gender | Was the researcher male or female? | N/A |
| 5. Experience and training | What experience or training did the researcher have? | ***P.6 research team and reflexivity:*** Furthermore, prior to data collection, the students received a half-day training in qualitative research methods, including qualitative interviewing techniques and principles of qualitative data analysis.  The research process was supervised by AC (GP, PhD) and AVD (MD, PhD), experienced researchers, with expertise in ID care). JW (MD, PhD), a professor of primary care and palliative care, and SO (MD, PhD), a GP and palliative care researcher. All supervisors have extensive experience with qualitative research. |
| *Relationship with participants* |  |  |
| 6. Relationship established | Was a relationship established prior to study commencement? | ***P.5 Recruitment and sampling***: Participants were primarily recruited through the researchers’ professional networks and relevant patient organizations.  ***P.6 Research team and reflexivity:*** The interviewers did not have prior relationships with participants. |
| 7. Participant knowledge of the interviewer | What did the participants know about the researcher? e.g. personal goals, reasons for doing the research | ***P.5 Recruitment and sampling***: Candidates received an invitation letter containing information about the study objectives, procedures, and voluntary participation |
| 8. Interviewer characteristics | What characteristics were reported about the interviewer/facilitator? e.g. Bias, assumptions, reasons and interests in the research topic | ***P.6 Research team and reflexivity***: Interviews were conducted by three final-year medical students as part of their master’s thesis for the degree of medical doctor, with every time one student conducting an interview and roles rotating across interviews. |
| **Domain 2: study design** |  |  |
| *Theoretical framework* |  |  |
| 9. Methodological orientation and Theory | What methodological orientation was stated to underpin the study? e.g. grounded theory, discourse analysis, ethnography, phenomenology, content analysis | ***P.4 Study design***: This study employed a qualitative research design, using individual in-depth interviews. The data analysis was conducted in accordance with a reflective thematic analysis using an inductive approach. |
| *Participant selection* |  |  |
| 10. Sampling | How were participants selected? e.g. purposive, convenience, consecutive, snowball | ***P.5 Recruitment and sampling***: To capture diverse perspectives, purposive sampling was used to enhance heterogeneity within the study population with respect to participant age, gender, professional experience (for the GPs), and type of caregiving relationship. Participants were purposively selected based on their direct experience with ACP and DNR decision-making for adults with moderate to profound ID. Given the specificity of these experiences and the in-depth nature of the interviews, the aim was to obtain rich, detailed accounts rather than a large sample size. |
| 11. Method of approach | How were participants approached? e.g. face-to-face, telephone, mail, email | ***P.5 Recruitment and sampling:*** Thirty-four candidates received an invitation letter containing information about the study objectives, procedures, and voluntary participation.  ***P.6: Data collection***: Interviews were conducted individually, either in person in their own home or GP practice, or via Microsoft Teams, and lasted 30–60 minutes. |
| 12. Sample size | How many participants were in the study? | ***P.5 Recruitment and sampling***: (…) resulting in a total sample of 11 participants |
| 13. Non-participation | How many people refused to participate or dropped out? Reasons? | ***P.7 Findings***: Out of 34 individuals invited to participate, 11 agreed to be interviewed, including six family caregivers and five GPs. Reasons for non-participation were systematically collected: 20 invitees did not respond, and among those who provided feedback, time constraints and limited experience with the target population were cited as reasons for declining participation. |
| *Setting* |  |  |
| 14. Setting of data collection | Where was the data collected? e.g. home, clinic, workplace | ***P.6: Data collection***: Interviews were conducted individually, either in person in their own home or GP practice, or via Microsoft Teams |
| 15. Presence of non-participants | Was anyone else present besides the participants and researchers? | N/A |
| 16. Description of sample | What are the important characteristics of the sample? e.g. demographic data, date | ***Table 1*** presents the demographic characteristics of the participants. |
| *Data collection* |  |  |
| 17. Interview guide | Were questions, prompts, guides provided by the authors? Was it pilot tested? | ***P.6: Data collection***: Two semi-structured interview guides were developed: one for general practitioners and one for family caregivers, with shared questions. The guides were informed by a literature review and refined through discussion with the team of experienced qualitative researchers, ensuring fully addressing the research question. Both interview guides are provided as Supplementary Material 2. |
| 18. Repeat interviews | Were repeat interviews carried out? If yes, how many? | ***P.6 Data collection***: no repeat interviews were conducted. |
| 19. Audio/visual recording | Did the research use audio or visual recording to collect the data? | ***P.6 Data collection***: Interviews were audio-recorded and transcribed verbatim |
| 20. Field notes | Were ﬁeld notes made during and/or after the interview or focus group? | ***P.7 Research team and reflexivity***: Throughout data collection and analysis, reflective notes were maintained. |
| 21. Duration | What was the duration of the inter views or focus group? | ***P.6 Data collection***: Interviews were conducted individually, either in person in their own home or GP practice, or via Microsoft Teams, and lasted 30–60 minutes. |
| 22. Data saturation | Was data saturation discussed? | ***P.5 Recruitment and sampling***: Data sufficiency was reached after eight interviews, when no substantially new codes emerged during the analysis. Three additional interviews were conducted to further explore variation and refine the emerging themes, resulting in a total sample of 11 participants.  ***P.18 Strengths and limitations***: While the sample size was limited, data sufficiency was achieved, suggesting that the data were adequate to capture the key perspectives relevant to the study objectives. |
| 23. Transcripts returned | Were transcripts returned to participants for comment and/or correction? | ***P.6 Data collection***: The research team did not return transcripts to participants for correction |
| **Domain 3: analysis and ﬁndings** |  |  |
| *Data analysis* |  |  |
| 24. Number of data coders | How many data coders coded the data? | ***P.7 Data analysis***: The first three authors double-coded the interviews, discussing coding to reach consensus. The first four transcripts were additionally double-coded by the supervising researcher, and all transcripts were reviewed by this researcher. |
| 25. Description of the coding tree | Did authors provide a description of the coding tree? | ***P.8 Findings*:** Table 2 presents the subthemes that were combined to form each overarching theme. Thematic analysis of the interview data resulted in the identification of six major themes, which are described in detail below: (1) limited preparedness and understanding of advance care planning, (2) the need for open discussion, (3) challenges in communication, (4) the importance of involving key stakeholders, (5) the role of timing, and (6) positive outcomes of these conversations. |
| 26. Derivation of themes | Were themes identiﬁed in advance or derived from the data? | ***P.7 Data analysis***: An **inductive** thematic analysis, as outlined by Braun and Clarke, was conducted collaboratively |
| 27. Software | What software, if applicable, was used to manage the data? | ***P.6 Data collection***: NVivo software was used for data management and coding. |
| 28. Participant checking | Did participants provide feedback on the ﬁndings? | ***P.6 Data collection***: The research team did not return transcripts to participants for correction, |
| *Reporting* |  |  |
| 29. Quotations presented | Were participant quotations presented to illustrate the themes/ﬁndings? Was each quotation identiﬁed? e.g. participant number | ***P.7-13 Findings***: All themes are illustrated by identified quotes |
| 30. Data and ﬁndings consistent | Was there consistency between the data presented and the ﬁndings? | ***P.7-13 Findings***: All findings are thoroughly grounded in the data |
| 31. Clarity of major themes | Were major themes clearly presented in the ﬁndings? | ***P.7-13 Findings***: Thematic analysis of the interview data resulted in the identification of six major themes, which are described in detail below: (1) limited preparedness and understanding of advance care planning, (2) the need for open discussion, (3) challenges in communication, (4) the importance of involving key stakeholders, (5) the role of timing, and (6) positive outcomes of these conversations. |
| 32. Clarity of minor themes | Is there a description of diverse cases or discussion of minor themes? | ***P.7-13 findings***: Minor themes are clearly described and illustrated by quotes. |
